# From mapping evidence to engaging voices: Paving the way for research on the social determinants of dementia

**DOI:** 10.1002/alz.71706

**Published:** 2026-07-31

**Authors:** Anouk Geraets, Daria Jensen, Scott Chiesa, Isabelle Foote, Laura Smith, David Llewellyn, Timothy Daly, Sebastian Walsh

**Affiliations:** ^1^ Department of Social Sciences University of Luxembourg Esch‐sur‐Alzette Luxembourg; ^2^ Clinic of Cognitive Neurology University Medical Center Leipzig Leipzig Germany; ^3^ Department of Neurology Max Planck Institute for Human Cognitive and Brain Sciences Leipzig Germany; ^4^ Institute of Cardiovascular Science University College London London UK; ^5^ Centre for Preventive Neurology Queen Mary University of London London UK; ^6^ University of Exeter Medical School, St Luke's Campus Exeter UK; ^7^ FLACSO Argentina Buenos Aires Argentina; ^8^ Cambridge Public Health University of Cambridge Cambridge UK

1

Evidence on the social determinants of dementia (SDOD), health‐related factors that affect dementia risk beyond medical, demographic, and individual lifestyle factors, is accumulating. A recent scoping review from some members of the Deep Dementia Phenotyping Network (DEMON) SDOD International Research Group mapped the current evidence across six SDOD domains, and concluded that there is clear and consistent evidence for some SDOD (e.g., education, air pollution, socioeconomic status, ethnicity), while evidence for others is still emerging (e.g., housing quality/stability), or lacking entirely (e.g., incarceration).^1^ Our findings highlight that, while education and air pollution stand out as key targets for public health interventions, additional research is needed for other less‐studied domains and to unravel the complex interactions between different determinants.

In response, T.D. (now a co‐author here) highlighted evidence of heterogeneity in the needs, attitudes, and priorities of different populations, policy‐makers, and other professionals toward dementia risk reduction policy.^2^ He argued that quantitative evidence should be synthesized with qualitative evidence^2^ from different groups, including members of the public^3^, ^4^, researchers,^5^ and policy‐makers^6^ to provide clear action points to reduce inequalities in brain health.^7^

Subsequently, we collectively organized an in‐person workshop open to all delegates at the Alzheimer's Research UK 2026 conference (of whom *n* = 30 from the United Kingdom, Germany and Luxembourg attended our workshop), to discuss pathways of evidence generation across five pre‐selected SDOD topics: (1) Co‐creating dementia prevention with the public, policy‐makers, and public health practitioners; (2) Women's health, gender, and sexual identity; (3) Food environment, eating behavior, and reward mechanisms; (4) Pathways from early‐life social determinants; and (5) Social determinants, ancestry, and gene–environment. These topics were selected based on an online survey sent out to the networks of the DEMON SDOD group (Tables S1–S2). Respondents of this online survey (*n* = 28) were asked to identify the most pressing research gaps in the SDOD field, indicate how they might contribute if their proposed gaps were selected as key future research directions, specify their preferred meeting format, and state whether they would be able to attend if the meeting was organized as a satellite event at the Alzheimer's Research UK 2026 conference. The major findings from the focus group discussions at the in‐person workshop are tabulated (Table 1). Delegates covered multiple fields, including public health, epidemiology, ethics, social sciences, genetics, lived experience, and patient and public involvement and engagement (PPIE). Most participants were academics, spanning early‐, mid‐, and senior‐career researchers, as well as research funders.

**TABLE 1:** Major findings from focus group discussions at an in‐person workshop on the social determinants of dementia (Manchester, UK, February 2026).

Parameter	Opportunities for research and policy	Challenges encountered	Priorities for future work
(1) Co‐creating dementia prevention with the public, policy‐makers, and public health practitioners	Need for more co‐ordination between ongoing initiatives, for example, discussing prevention at the community level.	“Dementia” discourse is tied to individual‐level episodes of sickness and solutions, casting population‐level concerns into the background.	Determine the utility of articulating different concepts to discuss population‐level approaches to dementia risk reduction (e.g., dementia, brain health, public health, etc.)
(2) Women's health, gender and sexual identity	Need for a whole‐system approach and a shift from a one‐size‐fits‐all to a more tailored, equitable approach.	Lack of representation from diverse groups. Shortage of evidence on specific risks and experiences. Female specific symptoms being dismissed or minimized, potentially leading to delayed diagnoses, suboptimal care, and psychological distress.	Mandate data reporting by sex, gender, or both. Harness data banks, cohort studies, and electronic healthcare records to explore hormonal impacts, comorbidities, symptomology and progression. Inclusive research practices and PPIE. Outreach via relevant community groups and events.
(3) Food environment, eating behavior, and reward mechanisms	Existing and emerging data sources offer underexploited potential to link diet objectively to cognitive outcomes. Natural experiments arising from historical events, rapid dietary transitions, and policy reforms provide quasi‐experimental leverage that is difficult to obtain otherwise.	Shortage of longitudinal studies with confirmed dementia endpoints. Use of cognitive proxies that limit causal inference. Historical datasets carry substantial confounding from concurrent social and political changes, while cohorts recruiting predominantly healthy participants restrict generalizability. The evidence base for specific nutrients and their optimal quantities remains weak and contested, and conventional dietary assessment tools introduce considerable measurement error.	Natural experiments should be embedded in existing cohorts and link historic cross‐national dietary datasets to dementia outcomes. Validated, objective dietary assessment tools capable of capturing intake in diverse populations are urgently needed.
(4) Pathways from early‐life social determinants	Need for a better understanding of the extent by which early‐life social determinants may cause or confound many of the associations commonly reported linking midlife risk factors and late‐life dementia.	Identifying direct and indirect pathways influencing late‐life dementia risk is extremely challenging. Scarcity of whole life course cohorts means that quantifying potential early‐life impact is difficult to achieve as data is often absent, lacking in granularity, or subject to recall or selection biases. In datasets that do exist, cohort effects plus homogenous populations limit real‐world understanding. Biomarkers used as proxies for subclinical damage in older cohorts are often employed in younger study samples without real understanding of whether these represent the same pathological processes.	Research must move past classic observational analyses and seek better causal inference techniques to tease out direct and indirect contributions of early‐life social determinants on late‐life outcomes. For example, natural experiments and cross‐context designs which can help to uncouple potentially unmeasured confounding structures from exposures. Better understanding and use of longitudinal changes in proxy blood and imaging biomarkers is essential to quantify early subclinical damage while minimizing risks of pre‐existing differences, attrition biases and reverse causations often seen in older cohorts. Need for better engagement with young people to gauge their understanding and attitudes to life course dementia prevention and population‐level prevention strategies.
(5) Social determinants, ancestry, and gene–environment.	Need for more diverse samples to conduct equitable research on social determinants and modifiable risk factors of dementia. There is a lack of data available for non‐European ancestries. Yet, there are several global biobanks reaching more advanced points in data collection. Time to consider how we can best carry out genetic studies focused on SDOD.	There is a lack of samples available from non‐European ancestries, especially in South Asian populations. There is a heavy reliance on public databases largely based on European ancestry samples for follow‐up functional analyses, for example, gene expression and epigenetic databases derived from tissue/cell cultures. Modifiable risk factors for dementia vary in prevalence between global populations but currently there is no reliable way to explore the genetic underpinnings of how that relates to dementia risk.	Need to create a working group to catalogue and harmonize available resources from non‐White ethnicity/non‐European ancestry participants, which would provide the foundation for larger studies, for example, transethnic GWAS and construction of polygenic risk scores to use for examining interactions with SDOD. More diversity of PPIE‐related activities to provide valuable contextual insights and improve recruitment of underrepresented populations.

Abbreviations: GWAS, genome‐wide association study; PPIE, patient and public involvement and engagement; SDOD, social determinants of dementia.

Across the five SDOD topics, opportunities for research and policy consistently emphasize the need for a more integrated, population‐based, system‐wide (funders, policy‐makers, researchers, clinicians, leaders, public, drug developers), and inclusive dementia prevention approach. Such an approach should actively engage diverse stakeholders and make better use of existing resources, including community partnerships and natural experiments. Challenges encountered across all areas include a lack of diverse and representative samples, especially beyond European ancestry populations, limited longitudinal and high‐quality data, reliance on weak proxies, and difficulty establishing causal relationships due to confounding and fragmented evidence. Additionally, dominant individual‐level framings of dementia, underrepresentation of marginalized groups, and insufficient attention to lived experiences (e.g., nutrition in people with dementia) and female‐specific risk factors (e.g., hormonal contributions) further hinder progress.

Priorities for future work converged on the need to improve data quality and inclusivity (e.g., diverse cohorts, sex/gender‐disaggregated data), apply stronger causal inference methods (e.g., as natural experiments and mechanistic models) and inclusive research practices (e.g., childcare support for study participants, open science/access journals, compensation from work), better leverage of existing data infrastructures (e.g., cross‐context designs, merging cohorts), and embed PPIE throughout research design, to advance research on SDOD and create a more integrated, population‐based, system‐wide, and inclusive dementia prevention approach.^8^

In conclusion, the multidisciplinary meeting at the ARUK (Alzheimer's Research United Kingdom) conference resulted in pertinent discussions among delegates with diverse backgrounds about the opportunities, challenges, and priorities of five pre‐selected topics in the SDOD field. However, most participants were involved in research and based in the United Kingdom, which highlights the need to actively involve individuals outside the research field to prioritize region‐specific next steps in research in the SDOD field.

## CONFLICT OF INTEREST STATEMENT

The authors have no competing interests to declare. Author disclosures are available in the Supporting Information.

## Supporting information



Supporting Information: alz71706‐sup‐0001‐SuppMat.docx

Supporting Information: alz71706‐sup‐0002‐ICMJE.pdf
