# Supplementary material for: From mapping evidence to engaging voices: Paving the way for research on the social determinants of dementia
Source: Alzheimers Dement. 2026 Jul 31;22(8):e71706. doi: 10.1002/alz.71706 (PMC13425619; doi:10.1002/alz.71706)
Supplement: Supplementary file 1 — Supporting Information: alz71706‐sup‐0001‐SuppMat.docx [file ALZ-22-e71706-s001.docx]

**Supplementary Material**

**From mapping evidence to engaging voices: Paving the way for research on the social determinants of dementia**

Anouk F.J. Geraets^a*^, Daria E.A. Jensen^b,c^, Scott T. Chiesa^d^, Isabelle F. Foote^e^, Laura J. Smith^e^, David J. Llewellyn^f^, Timothy Daly^g^, Sebastian Walsh^h^

^a^ Department of Social Sciences, University of Luxembourg, Esch-sur-Alzette, Luxembourg

^b^ Clinic of Cognitive Neurology, University Medical Center Leipzig, Leipzig, Germany,

^c^ Department of Neurology, Max Planck Institute for Human Cognitive and Brain Sciences, Leipzig, Germany

^d^ Institute of Cardiovascular Science, University College London, London, UK

^e^ Centre for Preventive Neurology, Queen Mary University of London, London, UK

^f^ University of Exeter Medical School, St Luke's Campus, Exeter, UK

^g^ FLACSO Argentina, Buenos Aires, Argentina

^h^ Cambridge Public Health, University of Cambridge, Cambridge, United Kingdom

**Table S1. Identification of research gaps and interests**

| **Primary Area of Research** | **In your personal opinion, what are the most pressing research gaps in the SDOD field that should be prioritized at these events? Broad responses are fine, but please be specific if possible.** | **If one of the research gaps highlighted above (or another area relevant to your field of expertise) was identified as a key future direction for our research, to what extent might you contribute?** |
| --- | --- | --- |
| Dementia Prevention | - understanding mechanisms / key drivers of the association with dementia risk - cross-country / cross-cultural comparisons | Both leading or contributing |
| Public health | We should move from identification (ie of problem areas) to solutions (e.g., what works, or how can it work) so individuals can benefit more quickly. | I would theoretically be interested in contributing to a research theme in this field if led by someone else |
| Cerebrovascular contributions to dementia | Lack of consideration of external systemic constraints to risk reduction (eg neighbourhood deprivation) | I would theoretically be interested in contributing to a research theme in this field if led by someone else |
| AD, dementia and other neurodegenerative diseases | Understanding how societal determinants of dementia influence environmental and behavioural factors that can theoretically be modified to reduce the risk of dementia. | I would theoretically be interested in contributing to a research theme in this field if led by someone else |
| Epidemiology of aging | - Mechanisms by which social determinants may influence brain aging and dementia risk (also testing direct/indirect effects) - Assess differential of vulnerability according to SDOD status - Targets for dementia prevention | I would theoretically be interested in contributing to a research theme in this field if led by someone else |
| Brain injury and dementia | I’m new to this area, but neighborhood deprivation seems important. Also metrics such as walkability of neighbourhood. | I would theoretically be interested in contributing to a research theme in this field if led by someone else |
| Mental health and dementia epidemiology | - Methodology: examining potential causal pathways from social determinants to dementia, including mediators - Examining the role of social determinants from earlier in life (e.g. childhood and adolescence) - A focus on low- and middle-income countries - Clustering of social determinants - The roles of social exclusion and social capital | I would theoretically be interested in contributing to a research theme in this field if led by someone else |
| Dementia Rehabilitation | Need for attention to needs and contexts of LMIC, and especially women's SDOD eh societal responses to menopause. | I would theoretically be interested in contributing to a research theme in this field if led by someone else |
| Neuroimaging; Neurogenetics; Mixed pathologies | Interactions of SDOD with genetic factors and how this may lead to heterogeneity clinically | I would theoretically be interested in leading a future research theme in this field |
| care; trauma and dementia | Identifying the most suitable data for estimating dementia in underserved groups robust systematic reviews PPI | I would theoretically be interested in leading a future research theme in this field |
| Social justice and equity in dementia and brain health research | Systems level research in dementia and brain health.  Clinical trials are usually “WEIRD” so how can we address this How can we ensure all people have equitable access to good brain health, including Indigenous people  Cognitive testing that accounts for culture and diversity | I would theoretically be interested in leading a future research theme in this field |
| SUD | Socialisation and sense of purpose/ community | I would theoretically be interested in leading a future research theme in this field |
| Socioeconomic deprivation | Global dementia research  Fair dementia assessments for diverse populations | I would theoretically be interested in leading a future research theme in this field |
| Genetic Epidemiology & Dementia Prevention | Initially outlining determinants that have weak to no evidence and discussing what kind of data could be used to answer these questions as the first step. Then brainstorm study designs for each determinant to set up different potential studies for the group to work on going forward. This is a similar format to how the frailty workshop was done and it worked really well.  I also think with the more supported determinants it could be important to look at creating combined scores that account for potential correlation between determinants and their shared/unique association with dementia.  In addition to looking at more trad epidemiological and public health, I think having a group with interest in looking at the impact of ethnicity (ancestry) and socioeconomic status on genetic dementia risk would also be relevant to the group and could be a way to involve more disciplines. There could also be scope to look at gene x environment interactions between social determinants and dementia risk. | I would be interested in contributing to traditional epidemiology studies, but could potentially contribute to leading a research theme on underrepresented groups in genetics and gene-environment interactions between dementia risk and social determinants |
| Social epidemiology of brain related health | Operationalization and standardization of SDOD in research designs. Data linkage between SDOD and healthcare data. | I would theoretically be interested in contributing to a research theme in this field if led by someone else |
| dementia diagnostics, prevention and lifestyle change and inclusive design and technology | Climate change | I would theoretically be interested in contributing to a research theme in this field if led by someone else |
| brain health and prevention - interventions | Employment / work place contributions | I would theoretically be interested in contributing to a research theme in this field if led by someone else |
| Risk factors of neurodegeneration focussing on air pollution | urban designs (access to green space, affordances of walking or cycling, social spaces, accessibility, noise and air pollution levels), changes in socioeconomic status throughout the lifespan (e.g., falling into poverty or gaining wealth - as epigenetic research seems to show that this affects health) | I would theoretically be interested in contributing to a research theme in this field if led by someone else |
| The Intersection of Social Characteristics, Language, and Cerebrovascular Aging. | An important research gap in SDOD is understanding how migration experiences impact cognitive health, including disrupted education, language challenges, and sociocultural adaptation. Education is another key area: while low formal educational attainment is a known modifiable risk factor for dementia, we need to clarify which types of learning matter most, whether different forms of cognitive stimulation vary in effect, and how the timing of learning across the life course influences dementia risk. | I would theoretically be interested in contributing to a research theme in this field if led by someone else |
| Neuroepidemiology | i) Life-course risk factors: more cohort studies are needed to clarify how early-life and mid-life exposures (education, nutrition, cardiovascular health, social isolation) interact to influence late-life dementia incidence risk. ii) Multimorbidity and frailty: the interplay between social determinants, comorbidity trajectories, and frailty is underexplored, particularly how coexistance of multiple diseases accelerate biological aging. iii) Diverse populations: Most evidence comes from high-income countries. Research in underrepresented and low-resource populations is needed to capture global heterogeneity in exposures and outcomes. | I would theoretically be interested in contributing to a research theme in this field if led by someone else |
| Health Psychology, Neuropsychology, Qualitative, Mixed methods | -Interactions between SDOD -Evidence from non-western countries -Inclusive research that recruits underrepresented groups -Harnessing multiple data sets, using real-world datasets -Evidence-based public health campaigns targeting SDOD throughout life course | I would theoretically be interested in contributing to a research theme in this field if led by someone else |
| Impact of adverse life experiences (violence and abuse) on risk of dementia | - Factors relating to social inclusion. - Occupation. | I would theoretically be interested in contributing to a research theme in this field if led by someone else |
| Lifecourse Epidemiology | 1) For each area in the scoping review where a lack of evidence has been identified, I think it will be important to first identify which we actually have the best prospect of robustly addressing. The creation of a shared document prior to the event which catalogues all cohorts that attendees have involvement in/knowledge of may be useful here as will help to inform what studies may actually be most feasible (e.g. it may identify that there a multitude of studies with data involving factors like occupation but a complete lack of others like incarceration or gender identity). The ones with the best data availability should then be prioritised and appropriate study designs to try to better get at causality should be discussed (triangulation, etc). 2) From a personal point of view there are two areas that I am particularly interested in. First, to what extent are currently recognised biological midlife factors (think Lancet Commission factors such as obesity, hypertension, cholesterol, etc) potentially confounded/explained by early-life social determinants (e.g. how much of the relationship between obesity and future dementia actually a legacy of children from low SES being far more likely to be obese). Second, there exists a strong association between childhood SES and future dementia, but it remains unclear how much of this is a direct effect (i.e. affecting neurodevelopment and therefore potentially immutable) vs how much is exerted indirectly by continued low SES and other related social factors into adulthood (and therefore potentially preventable at later ages).  3) There are also questions about how much of what we think are social determinants may in fact have intergenerational genetic underpinnings, although I know less about this (but think relevant and interesting). | I would theoretically be interested in leading a future research theme in this field |
| I work (with Seb Walsh, Carol Brayne, and others) on defining the concept and approach of "public brain health". | Co-create priorities for dementia prevention with the public and with policy makers & PH practitioners.  See Dykxhoorn, J., et al. https://doi.org/10.1186/s12889-022-13775-9.   They used a very interesting methodology: "Participants were mixed, divided into small groups, and asked to review the potential determinants list, identify any missing determinants through a gap analysis, and then rank each determinant according to two factors: (1) important and (2) amenable to change."  This should absolutely be applied to dementia prevention. Public health is not just about evidence. | I would theoretically be interested in leading a future research theme in this field |
| Influence of stress, diet, gut microbiome, and obesity on brain health in cohort and interventional studies | gender & sexual identity understudied and the US will not further contribute to fill this gab, therefore european researchers should aim including standatised gender questionaires and tracking the female hormonal cycle in studies. Further studies on the menopause transition time are needed.  food environment studies in relation to brain health are needed in relation to eating behaviour and reward mechanisms in contrast of just controlling studies with SES/education.  air pollution & physical environment are coming, however little is known. | I would theoretically be interested in leading a future research theme in this field |
| Brain health/Cognitive neuroscience | Lack of connection between academia/research and the real impact of SDOD on people | I would theoretically be interested in leading a future research theme in this field |
| Health; Migration; Socioeconomic inequality and Social Stratification; Wealth | lack of intersectional studies; lack of longitudinal studies to capture cumulative disadvantage; gendered care burden; digital inequality in brain health (unequal access to cognitive health tools for prevention); stigma & dementia; global crises aftermath and their associations with cognitive decline. | Depends on specific topic and availability. |
| Aging Research with a focus on Non-Pharmacological Interventions and Vulnerable Populations | Intersectionality and cumulative disadvantage | I would theoretically be interested in leading a future research theme in this field |

**Table S2. Format of meeting**

| **What do you think would be most constructive format for an event to identify the most pressing research gaps in the SDOD field and to spur new collaborative efforts to address these?** | **Oral Presentations / Q&A** | **'Fireside Chat' / Panel Discussions** | **'Sandpits' / Roundtables** | **Informal Drinks / Networking Reception** | **We are currently in early discussions with Alzheimer's Research UK regarding a potential SDOD satellite event at their annual conference in Manchester UK from 24th-25th February 2026. Would you be able to attend?** |
| --- | --- | --- | --- | --- | --- |
| 'Sandpits' / Roundtables (collaborative small group activities allowing expert speakers and earlier career researchers to come together to informally develop new ideas and collaborations) ;Oral Presentations / Q&A (expert speakers giving presentations addressing latest developments and future directions for research into SDOD);'Fireside Chat' / Panel Discussions (expert speakers holding structured/moderated discussion addressing latest developments and future directions for research into SDOD);Informal Drinks / Networking Reception (broadly unstructured event allowing researchers to organically meet and discuss interests and develop new ideas); | 1-2 hrs | Half Day | Half Day | Half Day | Yes (remotely/hybrid) |
| 'Fireside Chat' / Panel Discussions (expert speakers holding structured/moderated discussion addressing latest developments and future directions for research into SDOD);'Sandpits' / Roundtables (collaborative small group activities allowing expert speakers and earlier career researchers to come together to informally develop new ideas and collaborations) ;Oral Presentations / Q&A (expert speakers giving presentations addressing latest developments and future directions for research into SDOD);Informal Drinks / Networking Reception (broadly unstructured event allowing researchers to organically meet and discuss interests and develop new ideas); | 1-2 hrs | 1-2 hrs | 1-2 hrs | Half Day | Yes (remotely/hybrid) |
| 'Sandpits' / Roundtables (collaborative small group activities allowing expert speakers and earlier career researchers to come together to informally develop new ideas and collaborations) ;Oral Presentations / Q&A (expert speakers giving presentations addressing latest developments and future directions for research into SDOD);'Fireside Chat' / Panel Discussions (expert speakers holding structured/moderated discussion addressing latest developments and future directions for research into SDOD);Informal Drinks / Networking Reception (broadly unstructured event allowing researchers to organically meet and discuss interests and develop new ideas); | Half Day | 1-2 hrs | Half Day | 1-2 hrs | Yes (remotely/hybrid) |
| 'Sandpits' / Roundtables (collaborative small group activities allowing expert speakers and earlier career researchers to come together to informally develop new ideas and collaborations) ;'Fireside Chat' / Panel Discussions (expert speakers holding structured/moderated discussion addressing latest developments and future directions for research into SDOD);Oral Presentations / Q&A (expert speakers giving presentations addressing latest developments and future directions for research into SDOD);Informal Drinks / Networking Reception (broadly unstructured event allowing researchers to organically meet and discuss interests and develop new ideas); | Half Day | Half Day | Half Day | 1-2 hrs | Yes (remotely/hybrid) |
| 'Fireside Chat' / Panel Discussions (expert speakers holding structured/moderated discussion addressing latest developments and future directions for research into SDOD);'Sandpits' / Roundtables (collaborative small group activities allowing expert speakers and earlier career researchers to come together to informally develop new ideas and collaborations) ;Oral Presentations / Q&A (expert speakers giving presentations addressing latest developments and future directions for research into SDOD);Informal Drinks / Networking Reception (broadly unstructured event allowing researchers to organically meet and discuss interests and develop new ideas); | Half Day | 1-2 hrs | 1-2 hrs | Half Day | Yes (remotely/hybrid) |
| 'Fireside Chat' / Panel Discussions (expert speakers holding structured/moderated discussion addressing latest developments and future directions for research into SDOD);Oral Presentations / Q&A (expert speakers giving presentations addressing latest developments and future directions for research into SDOD);'Sandpits' / Roundtables (collaborative small group activities allowing expert speakers and earlier career researchers to come together to informally develop new ideas and collaborations) ;Informal Drinks / Networking Reception (broadly unstructured event allowing researchers to organically meet and discuss interests and develop new ideas); | 1-2 hrs | 1-2 hrs | 1-2 hrs | 1-2 hrs | Yes (remotely/hybrid) |
| 'Fireside Chat' / Panel Discussions (expert speakers holding structured/moderated discussion addressing latest developments and future directions for research into SDOD);'Sandpits' / Roundtables (collaborative small group activities allowing expert speakers and earlier career researchers to come together to informally develop new ideas and collaborations) ;Oral Presentations / Q&A (expert speakers giving presentations addressing latest developments and future directions for research into SDOD);Informal Drinks / Networking Reception (broadly unstructured event allowing researchers to organically meet and discuss interests and develop new ideas); | Half Day | Half Day | Half Day | 1-2 hrs | Yes (remotely/hybrid) |
| Oral Presentations / Q&A (expert speakers giving presentations addressing latest developments and future directions for research into SDOD);'Fireside Chat' / Panel Discussions (expert speakers holding structured/moderated discussion addressing latest developments and future directions for research into SDOD);'Sandpits' / Roundtables (collaborative small group activities allowing expert speakers and earlier career researchers to come together to informally develop new ideas and collaborations) ;Informal Drinks / Networking Reception (broadly unstructured event allowing researchers to organically meet and discuss interests and develop new ideas); | 1-2 hrs | 1-2 hrs | 1-2 hrs | 1-2 hrs | Yes (remotely/hybrid) |
| 'Fireside Chat' / Panel Discussions (expert speakers holding structured/moderated discussion addressing latest developments and future directions for research into SDOD);'Sandpits' / Roundtables (collaborative small group activities allowing expert speakers and earlier career researchers to come together to informally develop new ideas and collaborations) ;Oral Presentations / Q&A (expert speakers giving presentations addressing latest developments and future directions for research into SDOD);Informal Drinks / Networking Reception (broadly unstructured event allowing researchers to organically meet and discuss interests and develop new ideas); | Full Day | Half Day | Half Day | Half Day | Yes (remotely/hybrid) |
| 'Fireside Chat' / Panel Discussions (expert speakers holding structured/moderated discussion addressing latest developments and future directions for research into SDOD);'Sandpits' / Roundtables (collaborative small group activities allowing expert speakers and earlier career researchers to come together to informally develop new ideas and collaborations) ;Oral Presentations / Q&A (expert speakers giving presentations addressing latest developments and future directions for research into SDOD);Informal Drinks / Networking Reception (broadly unstructured event allowing researchers to organically meet and discuss interests and develop new ideas); | 1-2 hrs | Half Day | Multi-Day | 1-2 hrs | Yes (remotely/hybrid) |
| 'Fireside Chat' / Panel Discussions (expert speakers holding structured/moderated discussion addressing latest developments and future directions for research into SDOD);Oral Presentations / Q&A (expert speakers giving presentations addressing latest developments and future directions for research into SDOD);Informal Drinks / Networking Reception (broadly unstructured event allowing researchers to organically meet and discuss interests and develop new ideas);'Sandpits' / Roundtables (collaborative small group activities allowing expert speakers and earlier career researchers to come together to informally develop new ideas and collaborations) ; | Half Day | Half Day | 1-2 hrs | 1-2 hrs | Yes (remotely/hybrid) |
| 'Fireside Chat' / Panel Discussions (expert speakers holding structured/moderated discussion addressing latest developments and future directions for research into SDOD);'Sandpits' / Roundtables (collaborative small group activities allowing expert speakers and earlier career researchers to come together to informally develop new ideas and collaborations) ;Oral Presentations / Q&A (expert speakers giving presentations addressing latest developments and future directions for research into SDOD);Informal Drinks / Networking Reception (broadly unstructured event allowing researchers to organically meet and discuss interests and develop new ideas); | Multi-Day | Half Day | Full Day | 1-2 hrs | Yes (remotely/hybrid) |
| 'Sandpits' / Roundtables (collaborative small group activities allowing expert speakers and earlier career researchers to come together to informally develop new ideas and collaborations) ;Informal Drinks / Networking Reception (broadly unstructured event allowing researchers to organically meet and discuss interests and develop new ideas);'Fireside Chat' / Panel Discussions (expert speakers holding structured/moderated discussion addressing latest developments and future directions for research into SDOD);Oral Presentations / Q&A (expert speakers giving presentations addressing latest developments and future directions for research into SDOD); | 1-2 hrs | 1-2 hrs | Half Day | 1-2 hrs | Yes (remotely/hybrid) |
| 'Sandpits' / Roundtables (collaborative small group activities allowing expert speakers and earlier career researchers to come together to informally develop new ideas and collaborations) ;Informal Drinks / Networking Reception (broadly unstructured event allowing researchers to organically meet and discuss interests and develop new ideas);'Fireside Chat' / Panel Discussions (expert speakers holding structured/moderated discussion addressing latest developments and future directions for research into SDOD);Oral Presentations / Q&A (expert speakers giving presentations addressing latest developments and future directions for research into SDOD); | Full Day | Half Day | Multi-Day | 1-2 hrs | Yes (in-person) |
| 'Sandpits' / Roundtables (collaborative small group activities allowing expert speakers and earlier career researchers to come together to informally develop new ideas and collaborations) ;'Fireside Chat' / Panel Discussions (expert speakers holding structured/moderated discussion addressing latest developments and future directions for research into SDOD);Oral Presentations / Q&A (expert speakers giving presentations addressing latest developments and future directions for research into SDOD);Informal Drinks / Networking Reception (broadly unstructured event allowing researchers to organically meet and discuss interests and develop new ideas); | Half Day | Half Day | Half Day | 1-2 hrs | Yes (in-person) |
| Oral Presentations / Q&A (expert speakers giving presentations addressing latest developments and future directions for research into SDOD);'Sandpits' / Roundtables (collaborative small group activities allowing expert speakers and earlier career researchers to come together to informally develop new ideas and collaborations) ;'Fireside Chat' / Panel Discussions (expert speakers holding structured/moderated discussion addressing latest developments and future directions for research into SDOD);Informal Drinks / Networking Reception (broadly unstructured event allowing researchers to organically meet and discuss interests and develop new ideas); | 1-2 hrs | 1-2 hrs | Half Day | 1-2 hrs | Yes (in-person) |
| 'Sandpits' / Roundtables (collaborative small group activities allowing expert speakers and earlier career researchers to come together to informally develop new ideas and collaborations) ;'Fireside Chat' / Panel Discussions (expert speakers holding structured/moderated discussion addressing latest developments and future directions for research into SDOD);Oral Presentations / Q&A (expert speakers giving presentations addressing latest developments and future directions for research into SDOD);Informal Drinks / Networking Reception (broadly unstructured event allowing researchers to organically meet and discuss interests and develop new ideas); | 1-2 hrs | 1-2 hrs | Half Day | 1-2 hrs | Yes (in-person) |
| 'Fireside Chat' / Panel Discussions (expert speakers holding structured/moderated discussion addressing latest developments and future directions for research into SDOD);'Sandpits' / Roundtables (collaborative small group activities allowing expert speakers and earlier career researchers to come together to informally develop new ideas and collaborations) ;Oral Presentations / Q&A (expert speakers giving presentations addressing latest developments and future directions for research into SDOD);Informal Drinks / Networking Reception (broadly unstructured event allowing researchers to organically meet and discuss interests and develop new ideas); | Half Day | 1-2 hrs | Half Day | 1-2 hrs | Yes (in-person) |
| Oral Presentations / Q&A (expert speakers giving presentations addressing latest developments and future directions for research into SDOD);'Fireside Chat' / Panel Discussions (expert speakers holding structured/moderated discussion addressing latest developments and future directions for research into SDOD);'Sandpits' / Roundtables (collaborative small group activities allowing expert speakers and earlier career researchers to come together to informally develop new ideas and collaborations) ;Informal Drinks / Networking Reception (broadly unstructured event allowing researchers to organically meet and discuss interests and develop new ideas); | Multi-Day | Half Day | Half Day | 1-2 hrs | Yes (in-person) |
| Oral Presentations / Q&A (expert speakers giving presentations addressing latest developments and future directions for research into SDOD);'Fireside Chat' / Panel Discussions (expert speakers holding structured/moderated discussion addressing latest developments and future directions for research into SDOD);Informal Drinks / Networking Reception (broadly unstructured event allowing researchers to organically meet and discuss interests and develop new ideas);'Sandpits' / Roundtables (collaborative small group activities allowing expert speakers and earlier career researchers to come together to informally develop new ideas and collaborations) ; | 1-2 hrs | Half Day | Full Day | Half Day | Yes (in-person) |
| 'Fireside Chat' / Panel Discussions (expert speakers holding structured/moderated discussion addressing latest developments and future directions for research into SDOD);'Sandpits' / Roundtables (collaborative small group activities allowing expert speakers and earlier career researchers to come together to informally develop new ideas and collaborations) ;Oral Presentations / Q&A (expert speakers giving presentations addressing latest developments and future directions for research into SDOD);Informal Drinks / Networking Reception (broadly unstructured event allowing researchers to organically meet and discuss interests and develop new ideas); | 1-2 hrs | Half Day | Half Day | 1-2 hrs | Yes (in-person) |
| 'Sandpits' / Roundtables (collaborative small group activities allowing expert speakers and earlier career researchers to come together to informally develop new ideas and collaborations) ;'Fireside Chat' / Panel Discussions (expert speakers holding structured/moderated discussion addressing latest developments and future directions for research into SDOD);Informal Drinks / Networking Reception (broadly unstructured event allowing researchers to organically meet and discuss interests and develop new ideas);Oral Presentations / Q&A (expert speakers giving presentations addressing latest developments and future directions for research into SDOD); | Full Day | Half Day | Full Day | 1-2 hrs | Yes (in-person) |
| 'Sandpits' / Roundtables (collaborative small group activities allowing expert speakers and earlier career researchers to come together to informally develop new ideas and collaborations) ;'Fireside Chat' / Panel Discussions (expert speakers holding structured/moderated discussion addressing latest developments and future directions for research into SDOD);Informal Drinks / Networking Reception (broadly unstructured event allowing researchers to organically meet and discuss interests and develop new ideas);Oral Presentations / Q&A (expert speakers giving presentations addressing latest developments and future directions for research into SDOD); | Half Day | 1-2 hrs | Full Day | 1-2 hrs | Yes (in-person) |
| Oral Presentations / Q&A (expert speakers giving presentations addressing latest developments and future directions for research into SDOD);'Fireside Chat' / Panel Discussions (expert speakers holding structured/moderated discussion addressing latest developments and future directions for research into SDOD);'Sandpits' / Roundtables (collaborative small group activities allowing expert speakers and earlier career researchers to come together to informally develop new ideas and collaborations) ;Informal Drinks / Networking Reception (broadly unstructured event allowing researchers to organically meet and discuss interests and develop new ideas); | Full Day | Half Day | Half Day | Half Day | Yes (in-person) |
| 'Fireside Chat' / Panel Discussions (expert speakers holding structured/moderated discussion addressing latest developments and future directions for research into SDOD);'Sandpits' / Roundtables (collaborative small group activities allowing expert speakers and earlier career researchers to come together to informally develop new ideas and collaborations) ;Oral Presentations / Q&A (expert speakers giving presentations addressing latest developments and future directions for research into SDOD);Informal Drinks / Networking Reception (broadly unstructured event allowing researchers to organically meet and discuss interests and develop new ideas); | 1-2 hrs | 1-2 hrs | Half Day | 1-2 hrs | Yes (in-person) |
| 'Sandpits' / Roundtables (collaborative small group activities allowing expert speakers and earlier career researchers to come together to informally develop new ideas and collaborations) ;'Fireside Chat' / Panel Discussions (expert speakers holding structured/moderated discussion addressing latest developments and future directions for research into SDOD);Oral Presentations / Q&A (expert speakers giving presentations addressing latest developments and future directions for research into SDOD);Informal Drinks / Networking Reception (broadly unstructured event allowing researchers to organically meet and discuss interests and develop new ideas); | 1-2 hrs | Half Day | Multi-Day | 1-2 hrs | Yes (in-person) |
| Oral Presentations / Q&A (expert speakers giving presentations addressing latest developments and future directions for research into SDOD);'Fireside Chat' / Panel Discussions (expert speakers holding structured/moderated discussion addressing latest developments and future directions for research into SDOD);'Sandpits' / Roundtables (collaborative small group activities allowing expert speakers and earlier career researchers to come together to informally develop new ideas and collaborations) ;Informal Drinks / Networking Reception (broadly unstructured event allowing researchers to organically meet and discuss interests and develop new ideas); | Full Day | Full Day | Full Day | 1-2 hrs | No (but would consider attending a similar event in future depending on date/location) |
| Oral Presentations / Q&A (expert speakers giving presentations addressing latest developments and future directions for research into SDOD);'Sandpits' / Roundtables (collaborative small group activities allowing expert speakers and earlier career researchers to come together to informally develop new ideas and collaborations) ;'Fireside Chat' / Panel Discussions (expert speakers holding structured/moderated discussion addressing latest developments and future directions for research into SDOD);Informal Drinks / Networking Reception (broadly unstructured event allowing researchers to organically meet and discuss interests and develop new ideas); | Half Day | 1-2 hrs | Half Day | 1-2 hrs | No (but would consider attending a similar event in future depending on date/location) |
